# The Effect of Oxalic Acid and Citric Acid on the Modification of Wollastonite Surface

**DOI:** 10.3390/ma16247704

**Published:** 2023-12-18

**Authors:** Shaomin Lin, Weijie Wang, Linguang Wu, Mingfeng Zhong, Chenyang Zhang, Yaling Yu, Zhijie Zhang, Yunying Wu

**Affiliations:** 1 School of Materials Science and Engineering, Hanshan Normal University, Chaozhou 521041, China; 2 School of Materials Science and Engineering, South China University of Technology, Guangzhou 510641, China; 3 Chaozhou Branch of Chemistry and Chemical Engineering Guangdong Laboratory, Chaozhou 521041, China; 4 Guangdong Chaoshan Institute of Higher Education and Technology, Chaozhou 521041, China

**Keywords:** wollastonite, organic acid, modification, diffusion control

## Abstract

The modification mechanism of low-molecular-weight organic acids on a single-chain silicate mineral (wollastonite) was investigated through a leaching method. Solid and liquid samples were analyzed using atomic absorption spectrophotometer (AAS), X-ray diffraction (XRD), scanning electron microscope (SEM), and Fourier-transform infrared spectroscopy (FTIR). After 720 h of reaction, the results revealed that the dissolution concentration of Si (2200 μmol/L) in citric acid solution is more than that (1950 μmol/L) in oxalic acid. In the composite acids (citric acid and oxalic acid), the dissolution concentration of Si release from wollastonite reached the maximum value of 3304 μmol/L. The dissolution data of Si in wollastonite were fittingly described by the parabolic equation (Ct = a + b*t*^1/2^), with the highest correlation coefficients (R^2^ > 0.993), in the presence of the low-molecular-weight organic acids. The dissolution data suggested that the dissolution reaction process of Si was consistent with the diffusion-controlled model. Citric acid exhibited a higher affinity for attacking the (200) surface, while oxalic acid was prone to dissolve the (002) crystal face. The synergistic effects of oxalic acid and citric acid led to the weakening of the XRD diffraction peak intensity of wollastonite. When exposed to composite acids, the surface of wollastonite was covered with insoluble reactants that restricted the substance diffusion and hindered the reaction. This study offers valuable theoretical insights into the modification or activation of wollastonite by composite low-molecular-weight organic acids.

## 1. Introduction

**Figure 1:**
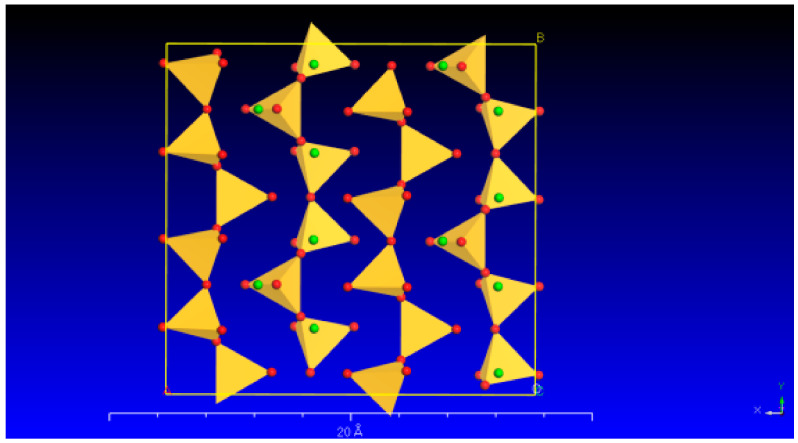
The crystal structure of wollastonite (the red and green balls represent O and Ca, respectively; yellow represents the [SiO_4_]).

Wollastonite is a single-chain silicate mineral with a chemical formula of Ca[Si_3_O_9_]. Its microstructure is commonly observed in the form of fibrous aggregates. The wollastonite crystal exhibits a repeating unit that comprises three [SiO_4_] tetrahedra. These tetrahedra combine to form a single-chain [Si_3_O_9_] structure along the b-axis. One of the tetrahedra’s edges aligns parallel to the chain’s extension, and the Ca cations occupy the spaces located between the parallel chains (Figure 1).

Wollastonite, known for its exceptional physical and chemical properties, such as excellent electrical and optical characteristics and its non-toxic nature, finds extensive applications in various industries [1,2,3]. In the ceramics industry, the addition of wollastonite into the raw materials not only reduces the firing temperature but also addresses the issue of excessive expansion during the sintering process [4]. Furthermore, the inclusion of high-aspect-ratio wollastonite significantly enhances product strength, improves the molding process, reduces energy consumption, elevates ceramic production yield and enhances overall product quality [5]. In conjunction with other silicate minerals, wollastonite exhibited remarkable chemical and thermal stability in glass–ceramic materials [6,7]. The plastic and rubber industries have recognized the utility of incorporating wollastonite in their processes [8,9,10]. Wollastonite, owing to its superior compatibility with plastics and rubber, has become widely adopted as a filler in the production process. When wollastonite is modified, it further enhances the chemical and thermal stability of the resulting products. Furthermore, important applications of wollastonite can also be found within the coatings industry [11,12,13]. When used as a filler in coatings, wollastonite contributes to improved physicochemical properties, particularly when incorporating high-aspect-ratio wollastonite that can produce a deflocculant effect, thus enhancing the coating’s anti-corrosion and anti-weathering capabilities. Additionally, wollastonite has gained significant usage in various fields, including construction [14,15,16], papermaking [17,18], and medicine [19,20].

However, wollastonite falls short in different industries with increasing new demands. Modification presents an opportunity to broaden its scope of application and enhance its properties. Various methods can be employed to modify wollastonite, including physical and chemical modification techniques [21,22,23]. Acid chemical modification is widely studied due to its simplicity of operation and low cost. The mechanism behind the acid chemical modification of silicate minerals involves the dissolution of skeleton elements, such as Si, from the mineral lattice [24,25,26]. There are reported works showing that the wollastonite can be leached and release Si in the presence of inorganic acid [27,28]. Zhang X. et al. [29] conducted experiments that revealed the ability of low molecular weight organic acids in silicate minerals to promote the dissolution of Si ions within the crystal structure of silicate minerals. This is mainly attributed to the formation of complexes between Si ions and organic acids. However, the effect of organic acids on the dissolution of wollastonite has seldom been studied.

In this work, wollastonite was treated with composite organic acids. The effect of composite organic acids on silicate minerals has received limited attention in prior research [30]. Therefore, the purpose of this article is to thoroughly investigate the modification effects of composite organic acids on wollastonite at room temperature. In particular, the investigation comprehensively examines the dissolution content of Si. and explores the structural changes in wollastonite. Moreover, a meticulous analysis of its modification mechanism will be carried out.

## 2. Materials and Methods

### 2.1. Materials

Both oxalic acid (C_2_H_2_O_4_·2H_2_O) and citric acid (C_6_H_8_O_7_·H_2_O) were bought from Fuchen Chemical Reagents Factory (Tianjin, China). Guangdong Changlong Porcelain Company Ltd. (Meizhou, China) supplied the wollastonite. The chemical composition of wollastonite is shown in Table 1. The median diameter (D50) of the wollastonite powder is 39.26 μm (provided by the supplier).

### 2.2. Experimental Procedure

Doubly de-ionized water (blank sample), 40 mmol/L oxalic acid, 40 mmol/L citric acid, and composite acids (20 mmol/L oxalic acid and 20 mmol/L citric acid) were added to four 200 mL polyethylene bottles. The solution was adjusted to pH = 4 with K_2_HPO_4_ (0.05 mol/L) and HCl (0.05 mol/L), and 2 mL of chloroform was added to stop microbial growth and prevent the oxalic acid and citric acid from degradation by microbes. Then, 4.0 g of the wollastonite powder was added to the prepared solution. After 10 min of vigorous stirring at 500 rpm/min, the solution was sealed and put in a biochemical incubator at 25 °C. At regular intervals of 8 h, the plastic bottles were moved and stirred at 500 rpm/min for 2 min. After 24 h, 48 h, 72 h, 96 h, 120 h, 480 h, 360 h, 480 h, 600 h, and 720 h of reaction, the mixtures were stirred for 3 min at the speed of 500 rpm/min, then centrifuged and filtered through 0.45 μm nylon filters. The liquid phase was rinsed in triplicate and kept for later use, while the solid phase was treated in an 80 °C vacuum drying oven for 48 h to remove moisture before being stored.

### 2.3. Characterization of the Solid

The loss on ignition of the wollastonite was determined by heating at 1000 °C for two hours. X-ray fluorescence (XRF, PANalytical Axios PW4400 spectrometer, Almelo, The Netherlands) was used to characterize the sample.

The wollastonite sample, after treatment with a citric, oxalic, or acid mixture for the longest time (720 h), was used for characterization in this paper. X-ray powder diffraction (XRD) patterns were obtained using an X’pertPro Panlytical diffractometer (PANalytical, Almelo, The Netherlands) operated at 40 kV and 40 mA with Cu Kα (0.15418 nm) filtered radiation. The XRD diffraction areas of wollastonite were calculated using the Origin 9.0 computer program. A Quanta 200 scanning electron microscope (SEM) equipped with a field emission gun was used for morphological analysis.

The Fourier transform infrared spectroscopy (FTIR) was carried out on the Vector 33 spectrometer produced by Bruker Corporation (Billerica, MA, USA) in the MIR range (4000–400 cm^−1^).

### 2.4. Characterization of the Dissolution Solutions

The concentration of Si was obtained by the Si molybdenum blue spectrophotometric method [31] with a visible light spectrophotometer (722N, Yoke Instrument Co., Ltd., Shanghai, China). In an acidic environment, silicic acid reacts with ammonium molybdate to produce a yellow silicon–molybdenum heteropolyacid. Subsequently, the yellow–molybdenum heteropolyacid complex is reduced into silicon–molybdenum blue with the aid of ammonium ferrous sulfate, and the silicon concentration is measured by spectrophotometry.

In order to investigate the mechanisms for the dissolution of Si from wollastonite, the dissolution data of Si extracted from sillimanite were fitted to the Parabolic diffusion equation (Equation (1)), the Elovich equation (Equation (2)), and the first-order equation (Equation (3)):

C_t_ = a + b*t*^1/2^ (1)

C_t_ = a + bln*t* (2)

C_t_ = a(1 − exp(−k*t*)) (3)

where C_t_ (μmol/L) is the concentration of the Si released from the mineral after reaction for *t* (hour), a and b are the constants of the kinetics equation, and k is the rate coefficient.

## 3. Results and Discussion

### 3.1. Dissolution Kinetics of Si in Different Organic Acids

**Figure 2:**
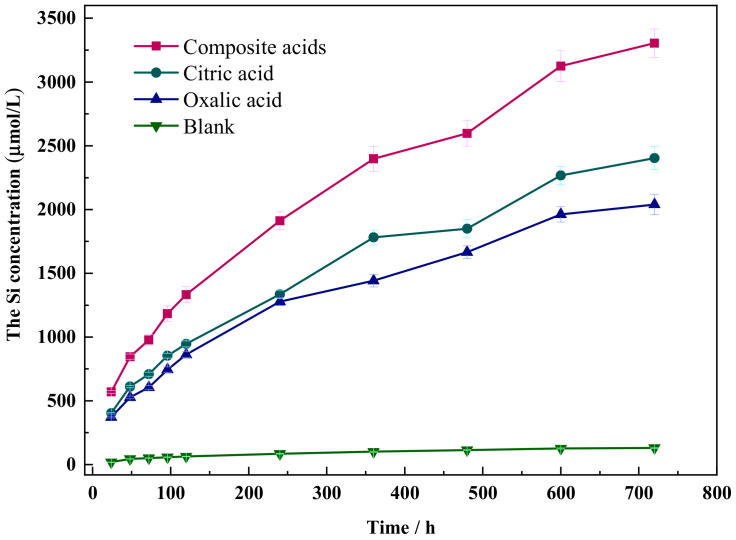
The dissolution curve of Si in different organic acids.

The dissolution curves of Si in wollastonite under various organic acids are depicted in Figure 2. The dissolution concentration of Si in citric acid or oxalic acid reaches the value of 2200 μmol/L and 1950 μmol/L, respectively, but the dissolution concentration of Si in the composite acid solution can reach up to 3304 μmol/L. Composite acid has the greatest dissolving impact, followed by citric acid, whereas oxalic acid has the weakest impact. The concentration of Si dissolved in wollastonite rose with time.

To fit the dissolution concentration data of Si in organic acids, Equations (1)–(3) were utilized. The dissolution data of Si in the blank samples did not fit the three equations well, and the coefficient of determination (R^2^) was less than 0.981 (Table 2). However, the results were markedly different when organic acids were present, with the best fit with the parabolic Equation (1) (R^2^ > 0.993). This suggests that the diffusion step controls the dissolution of Si in wollastonite. The release of Si resulted in the altered mineral volume per unit area, which also varied as $bt^{1/2}$. The parabolic rate constant b is proportional to the effective diffusion coefficient [32]. The higher the value of b, the easier the organic acid diffusion in the insoluble layer covering the mineral is, and the wollastonite is more vulnerable to attack.

### 3.2. Changes in the Crystal Structure of Wollastonite after Modification

**Figure 3:**
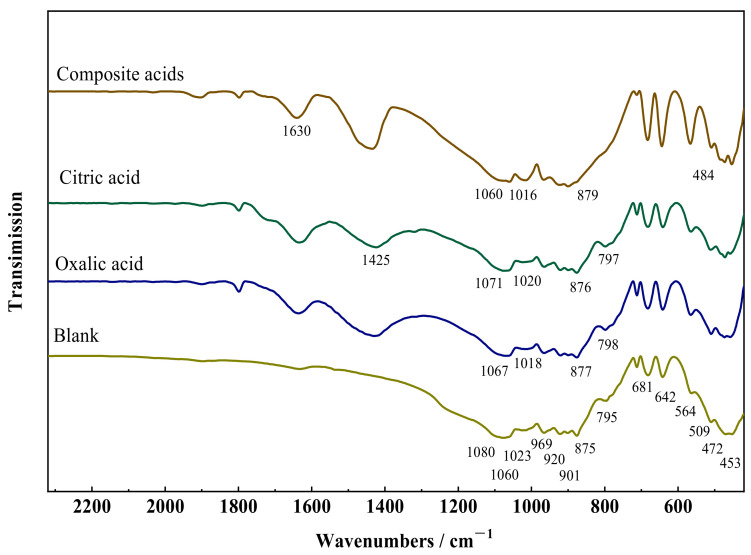
Fourier transform infrared (FTIR) spectra of wollastonite and LMWOAs treated wollastonite.

Figure 3 depicts the FTIR spectra of wollastonite after exposure to several organic acids. The stretching vibration of Si–O–Si and O–Si–O is responsible for seven absorption bands (1080, 1060, 1023, 966, 920, 901, and 875 cm^−1^) in the strong absorption range from 1100 to 850 cm^−1^. The absorption band (795, 681, and 642 cm^−1^) induced by the symmetric stretching vibration of Si–O–Si is connected with the absorption region spanning from 850 to 600 cm^−1^. The region exhibiting weak absorption from 600 to 400 cm^−1^ is made up of four absorption bands (564, 509, 472, and 453 cm^−1^) arising from the bending vibration of Si–O and the stretching vibration of Ca–O. The spectra show that the FTIR spectrum of wollastonite changed dramatically after treatment with organic acids. The vibrational absorption of organic groups in composite acids resulted in absorption bands corresponding to carboxyl group vibrations (COO–) at approximately 1630 cm^−1^ and 1425 cm^−1^.

The absorption peaks associated with the Si–O bond at 1080, 1060, and 1023 cm^−1^ in wollastonite shift towards lower wavenumbers after treatment with organic acids, whereas the absorption peaks related to the Si–O at 966, 920, 901, and 875 cm^−1^ shift towards higher wavenumbers. This phenomenon was attributed to the partial disruption of the crystal structure of wollastonite in organic acids. The following explanations can be given for these phenomena. Ca–O and Si–O are two primary chemical bond types in wollastonite crystals. The Ca–O bond is weaker and broken preferentially when exposed to organic acids. Bond strength and vibration absorption were enhanced when the Ca–O bond cleaves, as a large portion of the Si–O electronic cloud is concentrated close to the [CaO_6_]. The remaining portion of the Si–O electronic cloud in the Si–O–Si structure was reduced. The degree of the shift in the infrared vibration absorption peaks depends on how much the wollastonite crystal was damaged. The sequence of organic acid influence on the –O bond is as follows: complex acid > oxalic acid > citric acid, according to the infrared vibration data. This suggests that oxalic acid is more apt to weaken the Ca–O bond, thereby affecting the infrared vibration absorption of the Si–O bond.

**Figure 4:**
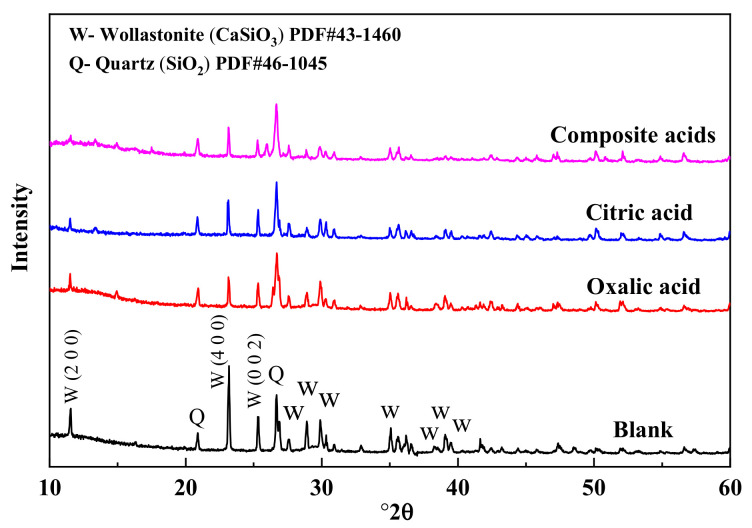
The XRD spectra of wollastonite in different organic acids.

The X-ray diffraction (XRD) patterns of wollastonite after being treated with various organic acids are depicted in Figure 4. The treatment with organic acids led to a decrease in the characteristic diffraction peak intensity of wollastonite (PDF#43-1460). Particularly, the diffraction peak intensities of (200) (I(200)) and (400) (I(400)) in the {100} crystal face family were significantly reduced, and that of the (002) plane (I(002)) was also diminished. There was very little difference in the diffraction peak intensity of quartz. The wollastonite crystal’s cleavage planes are {100} and {001}; {100} displays complete cleavage, while {001} exhibits comparatively modest cleavage. In mineral crystallography, cleavage is the breaking of a crystal’s lattice network under extreme stress in a specific direction. Cleavage planes constitute the weakest interlacing lattice planes, requiring relatively less energy for parallel crystal faces to rupture. The damage caused to a crystal face is more severe, and the corresponding diffraction peak intensity decreases significantly. The composite organic acids exhibit the most potent destructive ability. In this paper, the value of [I(200) + I(400)]/I(002) is used to discuss the destruction mechanisms of the wollastonite in the organic acids. As the value of [I(200) + I(400)]/I(002) increased, the {100} was less dissolved in wollastonite than {001}. According to the result of Table 3, the order for the value of [I(200) + I(400)]/I(002) was as follows: oxalic acid > composite acids > citric acid. Oxalic acid is easier to attack the crystal plane of {001} than citric acid, and citric acid is easier to dissolve the crystal plane of {100} than oxalic acid.

**Figure 5:**
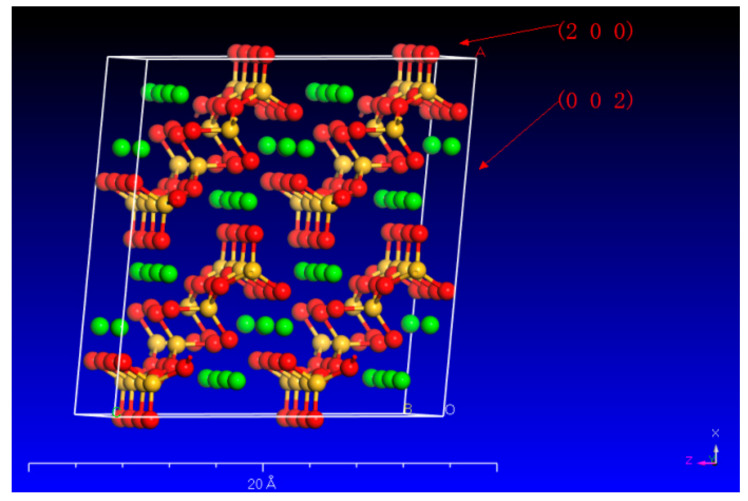
The crystal plane of (200) and (002) in wollastonite (the red, yellow, and green balls represent O, Si, and Ca, respectively).

Due to the diverse configuration of atoms (Ca and Si) on these surfaces, different organic acids have varying dissolving capacities on different wollastonite crystal surfaces. In particular, wollastonite’s (002) surface exhibits a higher crystal face density of Ca, resulting in oxalic acid being more effective in attacking the (002) crystal face than citric acid (Figure 5). On the other hand, the wollastonite’s (200) surface has more Si atoms, and citric acid demonstrates a greater capacity to dissolve Si than oxalic acid. This phenomenon suggested that different organic acids have different abilities to dissolve crystal surfaces.

### 3.3. Modification Mechanism of Wollastonite in Aqueous Citric Acid, Oxalic Acid or Mixed Acids

**Figure 6:**
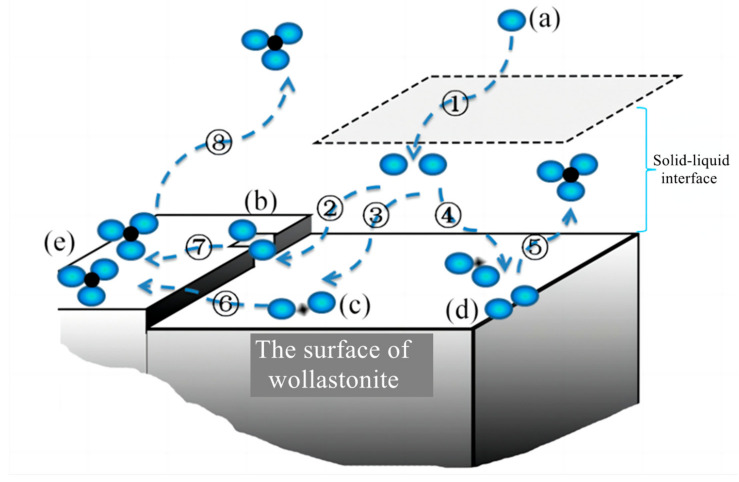
Mechanisms of ion sorption at the mineral–aqueous solution interface in the presence of organic acids. ①: the diffusion of (a) organic acid anions (blue spheres) from the liquid to the solid-liquid interface. ②, ③, and ④: the ions enter the solid-liquid interface and form surface complexes at various locations, such as (b) mineral steps, (c) surface depressions, and (d) edges. ⑤, ⑥, and ⑦: the extraction of elements (black spheres) in the mineral from the solid phase, forming (e) a complex and diffusing to the liquid phase. ⑧: the complex may adsorb onto the mineral surface.

The diffusion process involves multiple steps in the dissolving of silicate minerals: dissociation and diffusion of solutes in organic acid solutions, migration of ions or molecular at the solid–liquid interface, diffusion on the solid surface or in mesopores and micropores containing liquid phase (Figure 6). As organic acids enter the liquid film layer on the solid surface, they form surface complexes at various locations, such as mineral steps, surface depressions, and edges. As the number of organic acid molecules increases, the extraction of elements of the mineral from the solid phase forms a complex. This complex can diffuse back to the bulk phase through the liquid film layer, or it may adsorb onto the mineral surface.

According to an analysis of the Si dissolution curve, the dissolving mechanism of wollastonite is consistent with the diffusion-controlled model. This implies that the dissolution of wollastonite is governed by the diffusion process.

**Figure 7:**
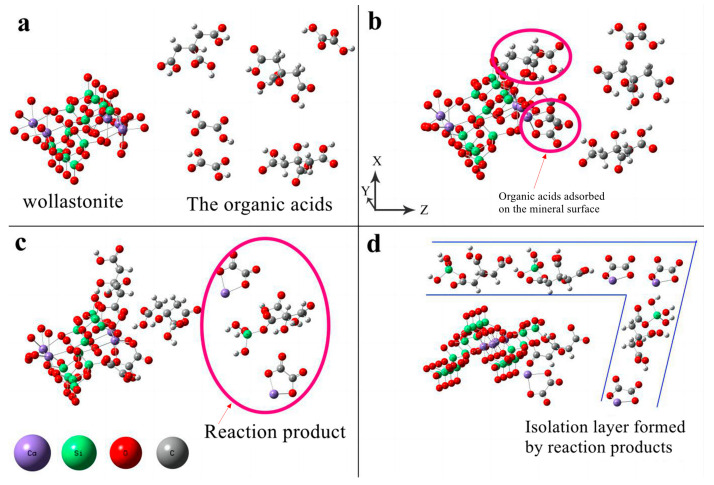
The schematic diagram of the dissolution mechanisms of wollastonite in the presence of composite organic acids. (**a**) organic acids move toward the wollastonite; (**b**) oxalic acid exhibits a higher affinity for adsorption on the Ca reaction site, while citric acid has a stronger tendency to be adsorbed on the Si reaction site; (**c**) The Si and Ca are extracted from the surface of wollastonite; (**d**) The isolation layer on the mineral surface formed by the insoluble products.

**Figure 8:**
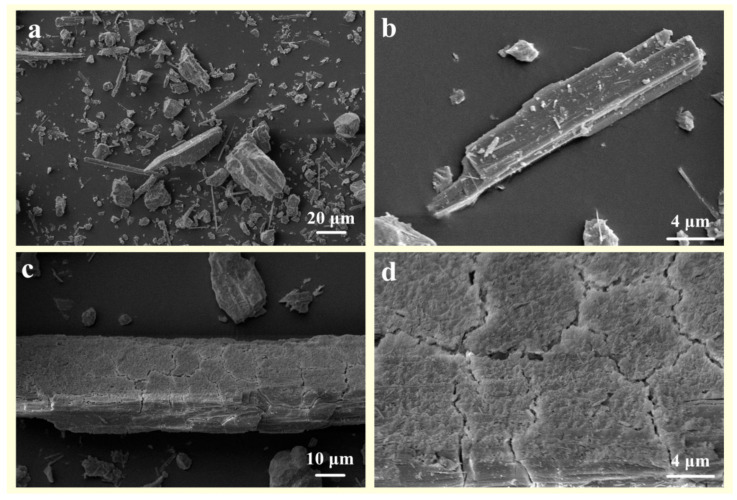
The SEM images of (**a**,**b**) raw wollastonite and (**c**,**d**) composite-organic-acid treated wollastonite.

In an ideal diffusion-controlled model, the process by which dissolved components of a mineral migrate to the diffusion layer (solid–liquid interface) on its surface, or the reactance in liquid migrates to the surface of the mineral, is referred to as the rate-limiting step. The diffusion layer forms a liquid-isolating membrane that surrounds the surface layer of silicate minerals. During the dissolution of wollastonite, ions are released and can form precipitates in a solution containing oxalic acid or citric acid. The reaction products adsorb on the mineral surface, forming an isolating layer that hinders the diffusion of organic acids to the reaction sites. The modification mechanism of composite organic acid on wollastonite is as follows (Figure 7):

(1) From the solution phase, organic acids move toward the solid–liquid interface. To be more precise, oxalic acid exhibits a higher affinity for adsorption on the Ca reaction site, while citric acid has a stronger tendency to be adsorbed on the Si reaction site (Figure 7a,b);
(2) Oxalic acid predominantly damages the (002) surface, while citric acid primarily attacks {100}. The synergistic effect of these two acids enhances the dissolution rate of wollastonite and leads to the destruction of more crystal faces (Figure 7c);
(3) As the reaction time increases, a considerable amount of Ca is leached from the wollastonite crystal. Both oxalic acid and citric acid have the potential to form substantial quantities of insoluble products. These materials create an isolation layer on the mineral surface (Figure 7d). As a result, the diffusion of reaction products into the liquid phase is impeded, resulting in a considerable decrease in the migration rate of organic acids to the reaction sites. Finally, the reaction rate gradually diminishes;
(4) In comparison to pure wollastonite (Figure 8a,b), the activation of wollastonite by organic acids is evident in the relative flattening of its sharp edges and corners (Figure 8c). Furthermore, the reaction products on the surface of wollastonite render it rough and uneven (Figure 8d).

## 4. Conclusions

The modification mechanism of both single and composite organic acids on wollastonite (a chain-like silicate mineral) was investigated. The dissolving process of Si in wollastonite under oxalic acid or citric acid is explored using a static experimental method and comparing studies of the solid and liquid phases. The results show that in the composite acids containing citric acid and oxalic acid, the concentration of Si can reach 3304 μmol/L. When utilizing single acids, the concentration of Si in the citric acid (2200 μmol/L) is greater than that in oxalic acid (1950 μmol/L) since the former is more prone to attacking the Si–O bond. Furthermore, the dissolution data of Si in wollastonite under the influence of organic acids are best fitted to the parabolic equation ($C_{t}=a+bt^{1/2}$) (R^2^ > 0.993), indicating that the reaction process is controlled by the diffusion step. The XRD diffraction peak intensity of wollastonite is the weakest under the influence of composite acids. In terms of single acids, oxalic acid is more prone to attacking the (002) surface, which has a higher density on the Ca face, while citric acid is more prone to dissolving the (200) surface, which has a higher density on the Si face. Under the synergistic influence of oxalic acid and citric acid, cracks occur on the surface of the wollastonite; insoluble reaction products accumulate on the mineral surface, and the reaction is inhibited. This investigation offers valuable theoretical insights into the modification of wollastonite by composite low molecular weight organic acids.

## Figures and Tables

**Table 1:** Chemical composition (wt%) of wollastonite.

Sample	SiO_2_	Al_2_O_3_	CaO	MgO	Fe_2_O_3_	Na_2_O	K_2_O	TiO_2_	P_2_O_5_	MnO	LOI
Wollastonite	53.82	0.87	41.2	1.53	0.21	0.12	0.21	0.05	0.03	0.03	1.93

LOI = Loss on ignition.

**Table 2:** The kinetic parameters of Si dissolution process under different organic acids (C_t_ (μmol/L) is the concentration of the Si released from mineral after reaction for t (h)).

The Solution	$C_{t}=a+bt^{1/2}$		$C_{t}=a+bInt$		$C_{t}=a(1-exp(-kt))$	
	b	R^2^	b	R^2^	k	R^2^
Blank	5.22	0.981	32.54	0.979	3.91 × 10^−3^	0.953
Oxalic acid	77.44	0.995	515.91	0.951	4.23 × 10^−3^	0.962
Citric acid	91.82	0.993	603.57	0.939	4.49 × 10^−3^	0.956
Complex acids	126.06	0.996	829.86	0.946	6.07 × 10^−3^	0.961

**Table 3:** The value of [I(200) + I(400)]/I(002) for wollastonite before and after treatment.

Organic Acids	Composite Acids	Citric Acid	Oxalic Acid
[I(200) + I(400)]/I(002)	2.05	1.86	2.33

## Data Availability

The data presented in this study are available on request.
